# FEMfuns: A Volume Conduction Modeling Pipeline that Includes Resistive, Capacitive or Dispersive Tissue and Electrodes

**DOI:** 10.1007/s12021-020-09458-8

**Published:** 2020-04-18

**Authors:** M. Vermaas, M. C. Piastra, T. F. Oostendorp, N. F. Ramsey, P. H. E. Tiesinga

**Affiliations:** 1 Department of Neuroinformatics, Donders Institute for Brain, Cognition and Behaviour, Radboud University Nijmegen, Nijmegen, the Netherlands; 2 Radboud University Nijmegen Medical Centre, Cognitive Neuroscience, Donders Institute for Brain, Cognition and Behaviour, Nijmegen, The Netherlands; 3 Department of Neurology and Neurosurgery, Brain Center Rudolf Magnus, University Medical Center Utrecht, Utrecht, The Netherlands

**Keywords:** Computational modeling, Finite element method, Electrical double layer, Dispersive tissue, Complete electrode model

## Abstract

Applications such as brain computer interfaces require recordings of relevant neuronal population activity with high precision, for example, with electrocorticography (ECoG) grids. In order to achieve this, both the placement of the electrode grid on the cortex and the electrode properties, such as the electrode size and material, need to be optimized. For this purpose, it is essential to have a reliable tool that is able to simulate the extracellular potential, i.e., to solve the so-called ECoG forward problem, and to incorporate the properties of the electrodes explicitly in the model. In this study, this need is addressed by introducing the first open-source pipeline, FEMfuns (finite element method for useful neuroscience simulations), that allows neuroscientists to solve the forward problem in a variety of different geometrical domains, including different types of source models and electrode properties, such as resistive and capacitive materials. FEMfuns is based on the finite element method (FEM) implemented in FEniCS and includes the geometry tessellation, several electrode-electrolyte implementations and adaptive refinement options. The Python code of the pipeline is available under the GNU General Public License version 3 at https://github.com/meronvermaas/FEMfuns. We tested our pipeline with several geometries and source configurations such as a dipolar source in a multi-layer sphere model and a five-compartment realistically-shaped head model. Furthermore, we describe the main scripts in the pipeline, illustrating its flexible and versatile use. Provided with a sufficiently fine tessellation, the numerical solution of the forward problem approximates the analytical solution. Furthermore, we show dispersive material and interface effects in line with previous literature. Our results indicate substantial capacitive and dispersive effects due to the electrode-electrolyte interface when using stimulating electrodes. The results demonstrate that the pipeline presented in this paper is an accurate and flexible tool to simulate signals generated on electrode grids by the spatiotemporal electrical activity patterns produced by sources and thereby allows the user to optimize grids for brain computer interfaces including exploration of alternative electrode materials/properties.

## Introduction

Stimulating and recording the brain by means of electrodes provides a versatile method to deepen our understanding of neural networks and their role in cognitive processes. Reconstructing the spatio-temporal distribution of neural current sources underlying electrophysiological data, such as electroencephalography (EEG) and electrocorticography (ECoG), assists in studying neural processes. Estimating the sources corresponds to solving the forward- and inverse problem.

The forward problem assumes a known source and solves for the electric potential in the brain. The inverse problem consists of estimating the source configuration underlying the recorded potential. The inverse problem requires solving the forward problem first and consequently the accuracy of the estimated sources will depend on the accuracy of the solution of the forward problem.

The Finite Element Method (FEM) is a suitable numerical method to solve the forward problem; it can incorporate the complex geometry of the head and allows for anisotropic conductivities, for example, to account for the laminar structure of cortex (Goto et al. 2010). FEM has been used to quantify various volume conduction effects, such as the influence of skull anisotropy (Marin et al. 1998), tissue inhomogeneities and anisotropies (Butson et al. 2007), and dispersive tissue properties (Grant and Lowery 2010).

A realistic description of the geometry and correct values of the electrical parameters of the biological tissues are essential to ensure an accurate forward model. The electrical conductivity and relative permittivity of biological tissues vary with frequency (i.e., they are dispersive, Gabriel et al. 1996; Miceli et al. 2017). However, volume conductor models used in bio-electricity generally consider only resistive currents, which is consistent with the so-called quasi-static approximation of Maxwell’s equations (Plonsey and Heppner 1967; Nunez and Srinivasan 2006), and capacitive, inductive and propagation effects are assumed to be negligible (Miceli et al. 2017). Whether the extracellular medium can be regarded as purely ohmic remains a topic of discussion (Gomes et al. 2016; Bédard and Destexhe 2017; Miceli et al. 2017; Logothetis et al. 2007).

A limited number of studies have addressed the effect of the presence of electrodes on the forward solution (Joucla and Yvert 2009; Butson and McIntyre 2005; Cantrell et al. 2007; Grant and Lowery 2010). The electric properties of electrodes are typically non-linear because of the properties of the current density distribution along its surface (Neuman 1998). In particular, when an electrode is immersed in an electrolyte, the charge carrier between the two materials changes from electronic in the metal to ionic in the electrolyte. As a result an electrical double layer forms on the external surface of the electrode where, in recording electrodes, a mix of faradaic (ohmic) and non-faradaic (capacitive) currents occurs depending on the magnitude of the potential difference across the interface (Richardot and McAdams 2002).

These ohmic and capacitive currents across the electrode have been implemented in FEM studies in a variety of set-ups, e.g., imposing faradaic currents (Joucla and Yvert 2009), non-faradaic currents (Butson and McIntyre 2005) or a parallel combination of the two (Cantrell et al. 2007; Grant and Lowery 2010). Models of recording electrodes, such as EEG, generally assume a simple point electrode model, while only a handful studies considers EEG forward models including the effect of electrode size and shunting (Ollikainen et al. 2000; Pursiainen et al. 2017).

In this study, we describe the workflow and capabilities of a volume conduction modeling pipeline FEMfuns (FEM for useful neuroscience simulations). The goal of this pipeline is to provide a Python-based framework centered around a general FEM toolbox, i.e., FEniCS (Alnæs et al. 2015; Logg and Wells 2010), to make forward models available, easily exploitable and adjustable for the neuroscience community. The volume conductor in FEMfuns can be described by resistive, capacitive and dispersive material properties. Furthermore, electrode interface effects can be flexibly added and the accuracy of the forward solution is described.

## Methods

In this work we conducted three different studies with the goal of demonstrating the capabilities of our FEMfuns pipeline. In all the studies Lagrangian FEM (Vorwerk et al. 2018) was applied to simulate the electric potential generated in a volume conductor by a known source. Both an internal dipolar source and an externally induced stimulating electrode are implemented. This can be useful considering the fact that the sensitivity of detecting bioelectric signals and the distribution in electrical stimulation are interchangeable (Malmivuo and Plonsey 1995), due to the reciprocity theorem (Helmholtz 1853; Rush and Driscoll 1969; Hu 2001).

### Forward Model

The electric potential *φ* generated in the brain can be computed through the quasistatic approximation of Maxwell’s equations (Plonsey and Heppner 1967). In our work, we considered two representations of the volume conductor, namely a purely resistive model and one that includes capacitive tissue properties. In the resistive version with primary current density ***J***_*p*_ (current produced by neuronal activity, e.g. a dipole, or from stimulating electrodes), ohmic currents are described in medium Ω with conductivity *σ* through the following equation:

1 $$  -\nabla\cdot(\sigma \nabla \varphi) = \nabla\cdot\boldsymbol{\mathrm{J}}_{p}, \quad\text{in } {\Omega}. $$

When taking into account the capacitive tissue properties, the quasi-static approximation of Maxwell’s equations does not hold anymore and the following frequency-dependent Poisson equation (Plonsey and Heppner 1967) has to be considered instead:

2 $$  -\nabla\cdot([\sigma(\omega) + j\omega\varepsilon_{0}\varepsilon_{r}(\omega)]\nabla \varphi) = \nabla\cdot\boldsymbol{\mathrm{J}}_{p}, \quad \text{in } {\Omega}, $$

where *j* is the imaginary unit, *ω* = 2*πf* is the angular frequency of the source, *ε*_0_ is the permittivity in vacuum (8.85 × 10^− 12^*F*/*m*) and *ε*_*r*_ is the relative permittivity. In case of a stimulating pulse or periodic currents generated by synchronous oscillations of neuronal circuits, a fast Fourier transform (FFT) is performed on the time series of the source. Then, the FEM is solved for each frequency separately and the signal in the tissue is reconstituted using the inverse FFT. This FEM-Fourier approach is comparable to several previous FEM studies (Butson and McIntyre 2005; Grant and Lowery 2010; Tracey and Williams 2011). It is essential to ensure the correct relationship between the real and imaginary part of an immittance, which is given by the Kramers-Kronig transforms (Van Gemert 1972; Bechhoefer 2011; Miceli et al. 2017; Bédard and Destexhe 2018). Both the medium (Van Gemert 1972; Gabriel et al. 1996) and the electrode interface impedance (Van Meirhaeghe et al. 1976; Macdonald and Urquidi-Macdonald 1990; Richardot and McAdams 2002) values we use satisfy the Kramers-Kronig relationship.

In both the resistive (1) and capacitive (2) scenario, a homogeneous Neumann boundary condition (BC) is applied on the exterior boundary *∂*Ω,

3 $$  \sigma\nabla \varphi \cdot \boldsymbol{n} = 0, \text{ on } \partial{\Omega}, $$

where ***n*** is the unit outer normal vector on *∂*Ω.

### The Finite Element Method for Solving the Forward Problem

Lagrangian FEM was used to solve the Poisson (1) (Logg et al. 2012; Larson and Bengzon 2013; Langtangen and Mardal 2016). The first step consists of deriving the so-called weak formulation of the elliptic partial differential equation (1) (Langtangen and Mardal 2016):

4 $$  a(u,v) = L(v), \forall u,v \text{ in } V \in H^{1}({\Omega}), $$

5 $$  \text{where } a(u,v) = {\int}_{{\Omega}} \sigma \nabla u \cdot \nabla v \text{ d}x $$

6 $$  \text{and } L(v) = {\int}_{{\Omega}} f v \text{ d}x, $$

where *H*^1^ is the first-order Sobolev space. The weak form can be heuristically derived by multiplication with a test function *v* ∈ *V* and subsequent partial integration. Reorganization of some terms and applying the homogeneous Neumann BC leads to (5) and (6).

In the second step, (4) is discretized yielding the following linear system:

7 $$  A u = b, $$

with $A_{ij} = {\int \limits }_{\Omega } \sigma \nabla \varphi _i \cdot \nabla \varphi _j \text { d}x$ and $b_{i} = {\int \limits }_{{\Omega }} f \varphi _{i} \text { d}x$, and {*φ*_*i*_}_*i*_ a set of basis functions.

Next, the linear system (7) is solved and the finite dimensional solution $u = {\sum }_{j} u_{j} \varphi _{j}$ is found.

In order to solve (2) with FEM, we had to deal with complex numbers (i.e., admittivity *y*), whose direct use was not yet implemented in FEniCS. In particular, we assembled complex numbers in a representation using real-valued coupled-PDEs. Starting from the strong formulation in the complex function space, i.e.,

8 $$  -\nabla\cdot\left( \left( y_{r}+j y_{j}\right) \nabla \left( \varphi_{r}+j \varphi_{j}\right)\right) = f_{r}+jf_{j} \quad\text{in } {\Omega}, $$

we split trial functions, test functions and the admittivity tensor into a real and imaginary part, *u* = *u*_*r*_ + *ju*_*j*_, *v* = *v*_*r*_ + *jv*_*j*_ and *y* = *y*_*r*_ + *jy*_*j*_, respectively, therefore considering the mixed space *W* = *V* × *V*. This results in a matrix doubled in linear size, composed of four blocks of the matrix created for the real version.

The weak form can again be derived by multiplying with the test function followed by partial integration, which for the left hand side gives a real part:

$$ \begin{array}{@{}rcl@{}} a_{r}(u, v) &=& {\int}_{{\Omega}} (y_{r} \nabla v_{r} \cdot\nabla u_{r}) - (y_{r} \nabla v_{j} \cdot\nabla u_{j})\\ &&- (y_{j} \nabla v_{r} \cdot\nabla u_{j}) - (y_{j} \nabla v_{j} \cdot \nabla u_{r}) \text{ d}x \end{array} $$

and an imaginary part:

$$ \begin{array}{@{}rcl@{}} a_{j}(u, v) &=& {\int}_{{\Omega}} (-y_{i} \nabla v_{j} \cdot\nabla u_{j}) + (y_{r} \nabla v_{j} \cdot\nabla u_{r})\\ &&+ (y_{j} \nabla v_{r} \cdot\nabla u_{r}) + (y_{r} \nabla v_{r} \cdot\nabla u_{j}) \text{ d}x \end{array} $$

Without an imaginary source, the right hand side weak form contains real part $L_{r}(v) = {\int \limits }_{{\Omega }} f_{r} v_{r} \text { d}x$ and imaginary part $L_{j}(v) ={\int \limits }_{{\Omega }} f_{r} v_{j} \text { d}x$.

#### Electrode-Electrolyte Interface

There are several ways to approximate the impedance that results from the electrode-electrolyte interface (Cantrell et al. 2007; Joucla and Yvert 2009). When recording or stimulating with an electrode, ideally no electrochemical reactions occur and hence all currents are capacitive. This regime can be modeled with a capacitance and an infinite transfer resistance at the (non-faradaic) interface of a stimulating or recording electrode. Richardot and McAdams (2002) empirically find that a standard capacitor does not describe the non-faradaic impedance accurately. This requires a pseudocapacitive constant phase angle impedance *Z*_*CPA*_:

9 $$  Z_{CPA} = K(j\omega)^{-\upbeta}, $$

where *K* = 1.57Ω*m*^2^*s*^−β^ and β = 0.91 are physical constants (Cantrell et al. 2007; Richardot and McAdams 2002).

Joucla and Yvert (2009) estimated the electrode-electrolyte interface impedance by fitting their FEM solutions to experimental data. In their model, the interface currents are described with faradaic reactions using a thin-layer approximation with a real valued surface admittance *y*_*k*_ (*S*/*m*^2^) expressed by the so-called Robin BC applied at the *k*-th electrode:

10 $$  -\sigma{\partial \varphi\over\partial \text{n}} = y_{k}(\varphi-\varphi_{\text{metal}_{k}}), \text{on }{\Gamma}_{{~}_{\mathrm{R}}}^{k},\quad k=0,1, \ldots $$

where $\varphi _{\text {metal}_{k}}$ is the electrical potential of the *k*-th electrode, ${\Gamma }_{{~}_{\mathrm {R}}}^{k}$ is its boundary.

Cantrell et al. and Grant and Lowery considered a constant phase angle impedance (9) and a charge transfer resistance set up in parallel. The double layer (Helmholtz 1879) is assumed to be 1 nm thick and the overpotential-independent charge transfer resistance can be described with the gas constant *R*, temperature *T*, number of electrons per molecule *n*, Faraday’s constant *F* and the exchange current *I*_0_. The appropriate values for these parameters are discussed in detail in Cantrell et al. (2007). The charge transfer resistance *R*_*CT*_ (Ohm) is defined in terms of these variables:

11 $$  R_{CT} = \frac{RT}{nFI_{0}}. $$

In our approach, we used Robin boundary conditions (10) to describe the interface, with its surface admittance *y*_*k*_ described by the pseudocapacitance (9) and charge transfer resistance (11) set up in parallel. Depending on the chosen *y*_*k*_, the interface processes can be described as faradaic, non-faradaic or a combination of the two.

In the case of recording electrodes, (10) needs to be used self-consistently, since for each electrode the value of *φ*_metal_ is unknown. We use the Lagrange multiplier method (Hansbo et al. 2005; Hansbo 2006; Amdouni et al. 2012) to impose *φ*_metal_ as the surface integral over the electrode in Eq. 10:

12 $$  \varphi_{\text{metal}} = \frac{1}{S}{\int}_{S} \varphi \text{ d}S. $$

#### Interface Weak Form

The weak form of the Robin BCs (10) is found multiplying trial function *u* by the test function *v* and integrating over the boundary:

13 $$ -{\int}_{\partial{\Omega}} \sigma\frac{\partial \varphi}{\partial n}v \text{ d}s = \sum\limits_{k}{\int}_{{{\Gamma}_{R}^{k}}}y_{k}(\varphi-\varphi_{\text{metal}_{k}}) \text{ d}s. $$

To allow for complex numbers, similarly to the capacitive Poisson (2), the test and trial functions are split in real and imaginary parts. The surface admittance *y*_*k*_ is split into interface conductivity *g* and interface susceptivity *b*, i.e. *y*_*k*_ = *g* + *ib*. Now the Robin BC can be written as:

14 $$ \begin{array}{@{}rcl@{}} {\int}_{{{\Gamma}_{R}^{k}}}y_{k}(u-\varphi_{\text{metal}_{k}})v \text{ d}s &=& {\int}_{{{\Gamma}_{R}^{k}}}(g+jb)((u_{r}+ju_{i})\\ &&-\varphi_{\text{metal}_{k}})(v_{r}+j v_{i} ) \text{ d}s \end{array} $$

Expanding this equation yield to the real part and imaginary parts:

15 $$ \begin{array}{@{}rcl@{}} &&\text{Real: }{\int}_{{{\Gamma}_{R}^{k}}}(g u_{r} v_{r} - g u_{i} v_{i} - b u_{i} v_{r} - b u_{r} v_{i}) \text{ d}s\\ &&+ {\int}_{{{\Gamma}_{R}^{k}}}(b \varphi_{\text{metal}_{k}} v_{i} -g \varphi_{\text{metal}_{k}} v_{r}) \text{ d}s \end{array} $$

16 $$ \begin{array}{@{}rcl@{}} &&\text{Imaginary: } {\int}_{{{\Gamma}_{R}^{k}}}(g u_{i} v_{r} + g u_{r} v_{i} + b u_{r} v_{r} - b u_{i} v_{i}) \text{d}s\\ &&- {\int}_{{{\Gamma}_{R}^{k}}}(g \varphi_{\text{metal}_{k}} v_{i} + b \varphi_{\text{metal}_{k}} v_{r}) \text{d}s \end{array} $$

The variational formulation *a*(*u*,*v*) = *L*(*v*) needs all integrals depending on the trial function *u* on the left hand side (*a*(*u*,*v*)) and the remaining integrals on the right hand side (*L*(*v*)). Thus, the real and imaginary integrals from the Robin BC are split into two parts, with subscripts *i* and *r* indicating the imaginary and real parts, respectively:

17 $$ a_{r}(u, v) = \sum\limits_{k} {\int}_{{{\Gamma}_{R}^{k}}} (g u_{r} v_{r} - g u_{i} v_{i} - b u_{i} v_{r} - b u_{r} v_{i}) \text{d}s, $$

18 $$ a_{i}(u, v) = \sum\limits_{k} {\int}_{{{\Gamma}_{R}^{k}}} (g u_{i} v_{r} + g u_{r} v_{i} + b u_{r} v_{r} - b u_{i} v_{i}) \text{d}s, $$

19 $$ L_{r}(v) = \sum\limits_{k} {\int}_{{{\Gamma}_{R}^{k}}} (g \varphi_{\text{metal}_{k}} v_{r} - b \varphi_{\text{metal}_{k}} v_{i}) \text{d}s, $$

20 $$ L_{i}(v) = \sum\limits_{k} {\int}_{{{\Gamma}_{R}^{k}}} (g \varphi_{\text{metal}_{k}} v_{i} + b \varphi_{\text{metal}_{k}} v_{r}) \text{d}s. $$

### Geometrical Models

In this study, three different geometries were used.

**Fig. 1:**
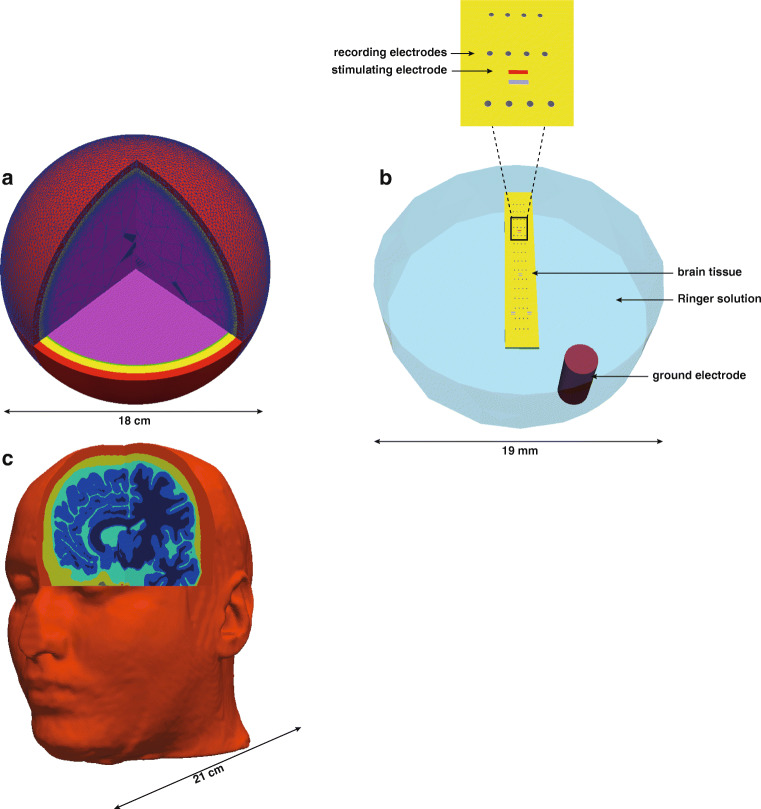
Geometrical models M1-M3. **a** Four-sphere head model (Naess et al. 2017), where the layers represent the different conductivities of the brain, CSF, skull and scalp compartments. **b** Bottom view of an experimental MEA set-up (Joucla and Yvert 2009) with a 200 *μ* m thick square slab of brain in the middle. The remainder of the cylinder is filled with Ringer’s solution (in blue). In the bottom right corner of the cylinder, the external cylindrical ground electrode (in purple) is positioned. On the tissue surface (in yellow) 60 distributed conical recording electrodes (in grey) and one square stimulating electrode (in red) are placed. Other square electrodes were not used for stimulation. The top panel displays a magnified region containing 8 recording and the stimulating electrode. **c** Realistic head model (Thielscher et al. 2015), segmented in scalp (red), skull (yellow), CSF (green), grey (light blue) and white matter (blue)

A four-layered sphere model (M1, Fig. 1a) representing the human head containing brain, cerebrospinal fluid (CSF), skull and scalp. The radii of the spheres are 9.0 cm, 8.5 cm, 8.0 cm and 7.9 cm, respectively (De Munck et al. 1993; Bauer et al. 2015; Naess et al. 2017). The mesh consists of about 12 million tetrahedra with a smallest inradius of 26 *μ* m and a largest of 0.5 cm. The script to generate the mesh used in this paper has been described in Naess et al. (2017).

The second geometric model (M2, Fig. 1b) corresponds to a multi-electrode array (MEA) set-up used in Joucla and Yvert (2009). The volume consists of a cylinder (diameter: 19mm, height: 5mm) filled with Ringer’s solution and a rectangular slab of neuronal tissue with dimensions similar to an embryonic mouse hindbrain-spinal cord. The MEA was positioned at the bottom of the tissue, with 60 conical recording electrodes (base diameter: 80 *μ* m, height: 80 *μ* m). The stimulating electrode was modeled as a rectangular surface (width: 60 *μ* m, length: 250 *μ* m) on the same MEA (Fig. 1b, inset in upper panel). An external cylindrical ground electrode (diameter: 2 mm, height: 4.3 mm) was represented by a cavity in the Ringer’s solution subdomain (Fig. 1b, purple cylinder positioned in the lower right region). We used the mesh that was generated by Joucla and Yvert using FEMLAB 3.1a (COMSOL AB, Stockholm, Sweden). It consists of 63,214 tetrahedra.

The third geometry is a realistic head model (M3, Fig. 1c) segmented in scalp, skull, CSF, grey and white matter (Ernie, provided in SimNIBS, Thielscher et al. 2015). For the grey and white matter, anisotropic conductivities estimated in SimNIBS (Thielscher et al. 2015) were used during the FEM calculations. The mesh contains approximately 4.2 × 10^6^ tetrahedra, where the inradius of each tetrahedra is below 1 mm.

### Simulation Set-Ups

The FEM simulations are performed using the open-source program FEniCS (Alnæs et al. 2015; Logg and Wells 2010). All simulations were done with Lagrange finite elements using the PETSc Krylov Solver with either the Conjugate Gradient method or generalized minimal residual method (GMRES) to solve the linear systems. Note however that a variety of other solvers is available.

Study 1: The linear system was numerically solved in the four-sphere head model (M1) using an average zero reference and compared to the analytical solution (Naess et al. 2017). The dielectric parameters *σ* and *ε*_*r*_ of the four layers were calculated using the four-term Cole-Cole expression (Gabriel et al. 1996) at 10 MHz (Table 1). This frequency serves as an example and other frequencies yield comparable results. Furthermore, since there is no agreement on the correct dispersive dielectric parameters (Gabriel et al. 1996; Miceli et al. 2017; Wagner et al. 2014), the values in this paper serve as an example. We solved the capacitive Poisson (2) with the homogeneous Neumann BC (3) on the outer surface. Dipoles were positioned at depths from 1 mm to 5 mm under the grey matter surface and oriented radially, tangentially or at a 45-degree angle. Dipoles were approximated with a positive and negative monopole of magnitude 100 *μ* A, at 1 mm distance from each other. Relative Differences ($RD = \frac {1}{N}{\sum }_{i=1}^{N} \frac {|\phi _{i} - \psi _{i}|}{{\max \limits } {|\psi |}}$), where *ϕ*_*i*_ is the numerical solution at location i, *ψ*_*i*_ the analytical solution and *N* the number of locations, were calculated on the surface between brain tissue and CSF at 32,400 evenly distributed locations to compare the analytical and numerical solutions. These locations on the brain surface represent ECoG point-electrodes.

**Table 1:** Isotropic conductivities and mean conductivities of the anisotropic white and grey matter (left column) (Opitz et al. 2011)

Name	*σ* (S/m)	*σ* (S/m) 10MHz	*ε*_*r*_ 10MHz
Grey	0.276	.29	320
White	0.126	.16	176
CSF	1.654	2	109
Skull	0.010	.04	36.8
Scalp	0.465	.2	362

Dielectric properties for tissues at 10MHz calculated using the four-term Cole-Cole expression (Gabriel et al. 1996). This frequency serves as an example and other frequencies yield comparable results. The frequency of biopotentials and stimulating electrodes is generally below 10 kHz

Study 2: The capacitive Poisson (2) was solved in M2, taking into account four combinations of capacitive dispersive material effects: a) capacitive tissue and a capacitive electrode interface surface admittance, b) capacitive tissue and a pseudocapacitance electrode interface surface admittance, c) dispersive tissue and a capacitive electrode interface surface admittance, d) dispersive tissue and a pseudocapacitance electrode interface surface admittance. A 200 *μ* s rectangular pulse was applied by the stimulating electrodes and decomposed in 50,001 frequencies (between 0 and 1/(2 dt) Hz, where dt is 10 *μ* s). In the dispersive case, the conductivity and permittivity values of the tissue and Ringer’s solution were calculated for each frequency using the four-term Cole-Cole equation (Eq (8), Gabriel et al. 1996). For the capacitive case, the conductivity and permittivity values were calculated using the four-term Cole-Cole equation at the average frequency of the pulse FFT. The ground and stimulating electrode surface admittance was implemented as a Robin BC (10). The pseudocapacitive case consisted of an equivalent impedance of a pseudocapacitance ((9)) and a charge transfer resistance ((11)) set up in parallel (Cantrell et al. 2007). The capacitive electrode case used the capacitance and resistance of the parallel pseudocapacitive circuit at the average frequency of the pulse FFT. Note that the capacitive reactance of the electrode remains dependent on frequency.

Study 3: As a proof of principle, Eq. (1) with BC (3) was solved in a realistic head model (M3). Tissue properties (Table 1 first column) were resistive and anisotropic. The dipole was tangentially and radially oriented with regard to the average normal of one of the electrodes at a distance of 1 mm.

### Implementation

**Fig. 2:**
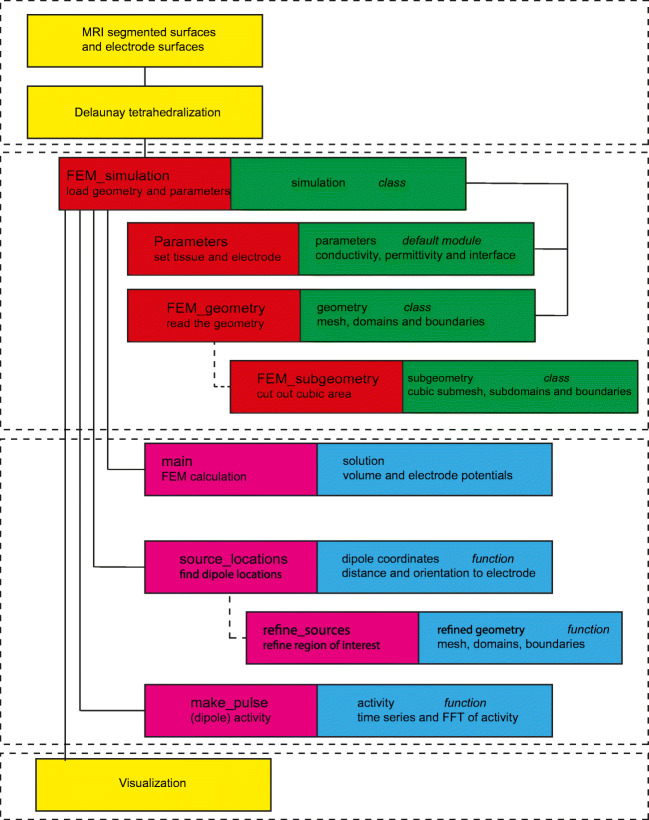
Sketch of the FEniCS pipeline (workflow goes from top to bottom). Red background indicates Python classes, green background the (main) output of the respective class. Purple background indicates functions in a class, blue the (main) output of the function and yellow indicates steps outside Python/FEniCS

The workflow of the simulation pipeline FEMfuns for the potential is visualized in a schematic overview (Fig. 2) and has the following steps:

1. Create the mesh (top yellow boxes in Fig. 2)
  - Define the different materials into separate subdomains and mark interfacial regions as boundaries
  - Convert geometry to FEniCS format
2. Choose simulation parameters for the (Parameters module in Fig. 2):
  - Type and location of source (e.g. electrode, monopole, dipole)
  - Capacitive/resistive/dispersive tissue (Table 1)
  - Electrode-tissue interface (10)
3. Create FEM simulation class instance (FEM_simulation in Fig. 2)
4. Run simulation (main in Fig. 2)
5. Visualize
6. Compare to analytical solutions (when possible)

Since only simple geometries can be created within FEniCS, other tools like gmsh (Geuzaine and Remacle 2008) or integrated realistic head models (Thielscher et al. 2015) should be considered for the generation of a tetrahedral mesh. The steps performed outside Python/FEniCS environment are indicated in yellow in Fig. 2. In this study we used both gmsh (Geuzaine and Remacle 2008) and SimNIBS (Thielscher et al. 2015). After creating the mesh, parameters regarding the materials, sources and electrodes need to be set (Fig. 2, in red are the Python classes and their use and in green the main output is shown). A subsection of the mesh can be cut out, creating a new smaller mesh. This is useful when solving for the potential in a whole head is not necessary (e.g. when using microelectrodes close to the source). The FEM_simulation class contains functions (indicated in pink, in blue is its main output) which set-up and solve the linear system. Dipole locations can be calculated (source_locations) based on distance and orientation with respect to an electrode, as well as inter-dipole distances. A procedure for mesh refinement in a region of interest is implemented, for example to study convergence, where a minimum cell inradius can be set. The stiffness matrix *A*_*ij*_ is computed in FEniCS with the main function. Based on the parameters that are chosen, resistive, dispersive or capacitive properties are used in the FEM calculation when calling main. In the frequency dependent analysis, a square pulse, alpha function or sine wave can be used as the activity waveform (e.g., make_pulse). Alternatively, a custom combination of frequencies can be given as input as well.

The Python code to obtain potentials from stimulating or recording electrodes, with three examples comparable to study 1-3, is available under the GNU General Public License version 3 at https://github.com/meronvermaas/FEMfuns.

## Results

### Study 1: Validation

**Fig. 3:**
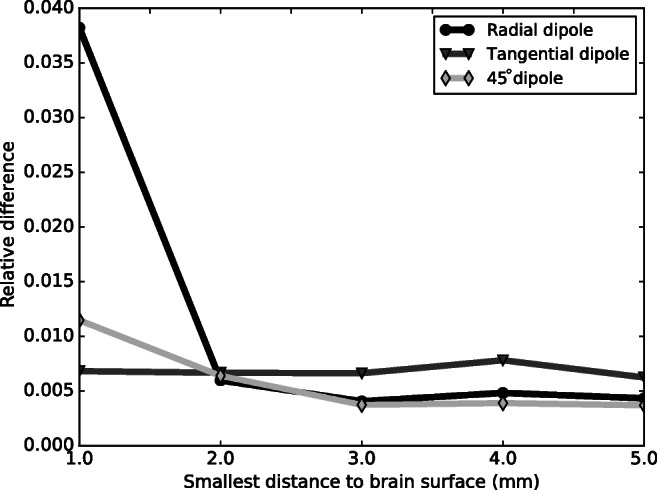
Relative difference (RD) of ECoG potentials calculated with the four-sphere model for a radial, tangential, and 45-degree dipole. Both capacitive and resisitive electrical properties of the tissue are taken into account via Eq. (2). The depth of the dipole below the cortical surface is varied (x-axis), error values are calculated at the surface of the grey matter

In study 1 we validated the accuracy of the numerical simulation in a four-sphere model with resistive and capacitive material properties (2) by comparing it with the analytical solution. Numerical and analytical potentials were compared on the outer surface of the sphere representing the cortical surface, which is comparable to an ECoG grid location. These observation points are close to the dipolar source (human cortical thickness is at most 5 mm), with the distances between the dipole and the surface ranging from 1 mm to 5 mm. The analytical solution adopted in this comparison consists of a series expansion (Naess et al. 2017) with 1000 terms. Only the conductivity in the analytical solution was changed into the admittivity, containing a real (conductance) and imaginary (susceptance) part. We computed the relative difference (RD) in the frequency domain at 10 MHz and visualized the results in Fig. 3. The RD values are typically small (below 0.04), whereas dipoles very close to the brain surface display the largest RDs. This is visible for the radial dipole in particular.

### Study 2: Dispersive Electrode and Tissue Implementation

**Fig. 4:**
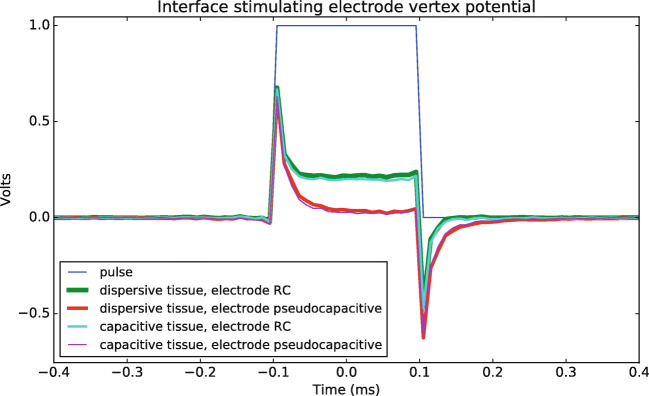
Waveform potential at a stimulating electrode vertex. Frequency decomposition of a stimulating square pulse allows to infer the effect of dispersive material (Gabriel et al. 1996). The dielectric properties of the electrode-electrolyte interface are calculated with a parallel pseudocapacitive component and resistance (Cantrell et al. 2007) or a capacitive component and resistance at the average frequency of the FFT. The original pulse and pulses at a vertex at the interface of the stimulating electrode are plotted and the combinations of electrode interface and tissue properties are indicated in the legend

The effect of capacitive and dispersive materials under voltage-controlled stimulation was investigated in this study and the results are visualized in Fig. 4. The applied square pulse is shown in blue. The line in red shows the voltage waveform at a vertex on the inside of the interface when the dielectric properties of the tissue are dispersive (conductivity *σ*(*ω*) and relative permittivity *𝜖*_*r*_(*ω*)) and the electrode interface is pseudocapacitive (a parallel pseudo-capacitance and charge transfer resistance). Dispersive tissue and a parallel RC electrode interface are shown in green, capacitive tissue and pseudocapacitive electrode interface in purple and capacitive tissue and RC electrode interface in light blue.

From Fig. 4 we notice that the voltage waveform at a vertex on the inside of the interface is deformed due to capacitive effects. The deformation of the potential at the vertex on the inside of the interface can mainly be explained by the time constant of the circuit representing the electrode interface. The two curves with a pseudocapacitive interface are overlapping (green and light blue curves), as well as the curves with an RC electrode interface (red and purple curves). Thus, in this geometry, the effect of the dispersive compared to capacitive tissue properties is negligible. Note the oscillations due to the Gibbs phenomenon which are minimized by increasing the padding around the pulse and the sampling frequency (Gibbs 1898). These results indicate substantial capacitive and dispersive effects due to the electrode-electrolyte interface when using stimulating electrodes.

### Study 3: Realistic Head Model

**Fig. 5:**
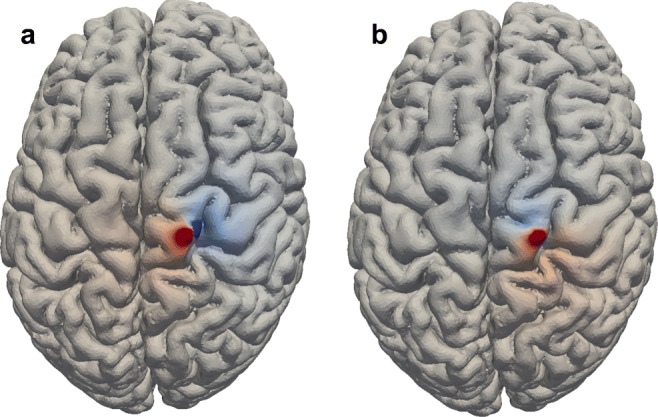
Grey matter region of potentials calculated in a realistic head model, with a tangential dipole left and radial dipole right. The dipole is placed 1 mm below the grey matter surface and the colormap is normalized

As a proof of concept, in the last example we solved (1) in a realistic head model with anisotropic resistive material properties. A radial dipole was positioned at 1 mm below the surface of one of the electrodes and the resulting potential distribution is shown in Fig. 5. In Fig. 5a the potential distribution resulting from a tangential dipole is displayed on the cortical surface. The transition from the positive to the negative potential can be observed clearly on the cortical surface. Figure 5b illustrates the potential distribution of a radial dipole on the cortical surface. The different polarity of the gyrus and the sulcus due to the relative angle of the surface with the dipole can be observed.

## Discussion

The purpose of this paper is to describe and introduce a Python-based framework centered around FEniCS for FEM forward calculations in electrophysiological recordings. We developed FEM scripts which allow neuroscientists to compute both resistive and frequency dependent capacitive material properties. Using three geometries, we show examples of the use of the FEMfuns pipeline. The main novelty that is presented here is the possibility to easily include electrode and capacitive material effects.

In study 1, we looked at dipole positions at several depths in a four-sphere head model and visualized the error at the surface directly on top of the brain, where ECoG grids are positioned (results in Fig. 3). The accuracy of the *capacitive* Poisson (2) was calculated, which expands on the original approach where the *resistive* Poisson (1) is solved on top of the scalp (Naess et al. 2017). With a tetrahedron inradius of 26 *μ* m at the region of interest, accurate numerical estimates were achieved with relative error values below 0.04. The RDs for dipoles very close to the brain surface are the highest. The two monopoles that were used to approximate a dipole need to be sufficiently close to each other. If the distance between the dipole and the recording location is much larger than the distance between the monopoles, the dipole will be more accurately approximated. Overall, these results demonstrate that dispersion effects can be accurately modeled.

Electrode effects have been studied extensively in stimulation studies (Robinson 1968; Butson and McIntyre 2005; Cantrell et al. 2007; Grant and Lowery 2010). Because of the reciprocity theorem, stimulating electrodes are useful models for recording electrodes as well. Non-linear behavior of the electrode-electrolyte interface is expected at high frequencies and at low frequencies provided that the applied signal amplitude is high (Richardot and McAdams 2002). Recording electrodes are unlikely to show major non-linear effects because the charge transfer resistance, Eq. (11), dominates the interface impedance.

In study 2, dispersion effects of the electrode interface (Cantrell et al. 2007) and the tissue properties (Gabriel et al. 1996) were examined in the stimulating electrode configuration (Fig. 4). The shape of the simulated voltage waveforms is comparable with dispersion effects reported in the literature (Butson and McIntyre 2005; Grant and Lowery 2010). As the driving potential amplitude and frequency increase, the dispersion effect becomes more noticeable (Cantrell et al. 2007). The interface impedance acts as a high-pass filter in study 2 (i.e., the interface time constant is short compared to the time period of the input waveform) (Irwin and Nelms 2010). Thus, the charging and discharging of the capacitance is faster than the change of the input waveform.

In our example, due to the small surface area of the recording electrodes, the interface impedance is high (e.g., 400 kΩ at 100 Hz and 6 kΩ at 10000 Hz, using the pseudocapacitive interface impedance). This resulted in negligible interface (capacitive or dispersive) effects of the recording electrodes and it is in line with previous literature which shows minimal effect of the recording electrode (Moulin et al. 2008; Joucla and Yvert 2009; Nelson and Pouget 2010). However, if larger electrodes are used, interface effects due to the recording electrode can be observed (Ollikainen et al. 2000; Pursiainen et al. 2017).

As a proof of principle (study 3), the forward solution was computed in a realistic head model with radial and tangential dipoles. The four-sphere model simulations demonstrated that numerical errors are negligible if tessellation is sufficiently fine. With no analytical solution available in the realistic head-model, convergence can be checked using adaptive refinement while monitoring percent change between the solutions.

Other open-source pipelines for solving the EEG and/or the magnetoencephalography (MEG) forward problems with FEM, or simulating electric stimulations are available (Hagen et al. 2018; Neymotin et al. 2019). Fieldtrip (Oostenveld et al. 2011), for example, is a MATLAB software toolbox for MEG, EEG, iEEG and NIRS analysis, that includes functions aiming at solving the EEG forward problem with FEM. Fieldtrip internally calls the C++ open source library called SIMBIO (Vorwerk et al. 2018, https://www.mrt.uni-jena.de/simbio/). To the best of our knowledge, in Fieldtrip it is not possible to simulate electrical stimulation and neither is it possible to easily change the properties of the electrodes. Another open-access tool dealing with solving partial differential equations in neuroscience is duneuro (Nüßing et al. 2019). Duneuro is an open-source C++ software library based on the DUNE library and its main features include solving the EEG (Engwer et al. 2017) and MEG (Piastra et al. 2018) forward problem and providing simulations for brain stimulation. There are Python and MATLAB wrappers which extend the usability of the software to a broader audience. In the present implementation of duneuro, neither the capacitive model nor the electric properties of the electrodes are incorporated in the workflow. A further example of open-source tools dealing with simulations in neuroscience is SimNIBS (Windhoff et al. 2013), whose aim is to provide an easy-to-use pipeline for conducting brain simulations with FEM in realistically shaped head models. SimNIBS is limited to brain simulations and it is not flexible for adjusting the type of electrode or adding capacitive material properties.

The main limitations of the pipeline concern mesh generation, which currently needs to be done in external software such as FSL (Jenkinson et al. 2012) or gmsh. Segmentation of the different head materials was not addressed in the current study. Furthermore, currently only tetrahedral mesh elements can be used in FEniCS. While they can fit the complex geometry of the brain better, it requires several non-trivial steps to convert the hexahedral voxels of an anatomical MRI.

A further limitation concerns the time needed for running the simulations. Depending on the geometry, tissue type and electrode implementation, the linear system that needs to be solved can become very large. In this study, the calculation time was around 1.5 hours per simulation for the four-sphere model (M1), 6 s per simulation in the FEM-Fourier example (M2), and under 2 minutes in the realistic head model with anisotropic tissue properties (M3) running on a Intel(R) Xeon(R) CPU E5-2640 v3 @ 2.60GHz processor. Furthermore, to achieve the desired convergence rate and accuracy, the solver, preconditioner, number of iterations and/or convergence tolerance need to be adjusted. For example, when using capacitive tissue, the Conjugate Gradient method will not converge, while GMRES will.

A further advantage of the FEMfuns pipeline, is that it is easy to control factors affecting the convergence. Furthermore, a variety of families (e.g., Discontinuous Lagrange, Nedelec, Raviart-Thomas) and degrees (linear, quadratic or higher) of elements are supported in FEniCS. This means that the pipeline can also be used in combination with neuron simulation software to provide extracellular potentials.

The results of this study have shown the first open-source, easy-to-use and flexible pipeline, allowing for the simulation of multiple material compartments in volume conductor models with as many compartments as needed (e.g., an arbitrary amount of electrode volumes can be used). Resistive, capacitive and dispersive tissue properties can be used and different types of electrode are implemented. Furthermore, the Python code can be easily adjusted and extended to meet the users needs.

## Information Sharing Statement

The FEMfuns code for using the new methods is publicly available at https://github.com/meronvermaas/FEMfuns and is licensed under the General Public License (GPL) v3. Documentation is provided at github, for support please contact the first author.
